# Temporal and spatial dynamics of the emerald ash borer invasion in Connecticut as shown by the native digging wasp *Cerceris fumipennis* (Hymenoptera: *Crabronidae*)

**DOI:** 10.3389/finsc.2023.1179368

**Published:** 2023-05-15

**Authors:** Claire E. Rutledge, Robert E. Clark

**Affiliations:** ^1^ Connecticut Agricultural Experiment Station, New Haven, CT, United States; ^2^ EcoData Technology, Plantsville, CT, United States; ^3^ Department of Entomology, Washington State University, Pullman, WA, United States

**Keywords:** biosurveillance, invasive species, *Agrilus Planipennis*, monitoring, temporal dynamics, spatial dynamics

## Abstract

Detecting and monitoring populations of the invasive emerald ash borer (EAB) is crucial to successful management of the pest and evaluation of its ecological impacts. However, the beetle’s cryptic habit makes accurate monitoring costly and time-consuming. Biosurveillance takes advantage of the foraging effort of a predatory wasp *Cerceris fumipennis* (Hymenoptera: Crabronidae). This native, solitary, ground-nesting hunting wasp hunts adult buprestid beetles to provision its brood cells. By intercepting the hunting wasps, we can learn which species of buprestids are in the surrounding forest. The resulting data provides information on the presence and relative abundance of invasive buprestids like EAB which can supplement other monitoring efforts. In this paper we share results of ten years of biosurveillance surveys of the EAB in Connecticut. Among 112 sites, we observed EAB populations; from first detection, through the population peak and then through to the population crash, matching patterns observed in other regions of the United States. We also observed the spread of the EAB relative abundance as it moved through the state following an invasion front starting in New Haven, Co. The average time from first detection to population crash was nine years. On average, populations peaked three years after first detection, and remained at peak levels for three to four years. Population decline was gradual and took another three to four years. Notably, no evidence of a second introduction to Connecticut was seen with proportional abundance increasing over time after expanding outward from the introduction point. These results corroborate other traditional monitoring efforts in the eastern U.S. and provide independent validation of predicted population dynamics in ash stands.

## 1 Introduction

Emerald ash borer (*Agrilus planipennis* Fairmaire) is native to Far Eastern Asia and was first detected in North America in 2002 (1). Emerald ash borer (EAB) is a phloem-consuming buprestid beetle that feeds on trees the genus *Fraxinus* (Oleacea). In its native range, the beetle is considered a secondary pest, where it rarely infests ash trees that are healthy and instead thrives in trees with weakened anti-herbivore defenses resulting from decadence, disease, or drought (2). EAB’s feeding strategy is similar to North American species of *Agrilus*, such as *Agrilus anxius* Gory and *Agrilus bilineatus* (Weber), whose larvae typically feed on weakened hosts in the birch and oak families (3, 4). However, healthy trees of North American species of *Fraxinus* are successfully attacked by EAB (5). Emerald ash borer has little competition for healthy ash phloem in North America, and is not known to have any native, specialist natural enemies. Lack of bottom-up control by plant defenses and top-down control by natural enemies is thought to contribute to the exceptionally fast and devastating spread of this invasive insect across North America (1).

**Figure 1:**
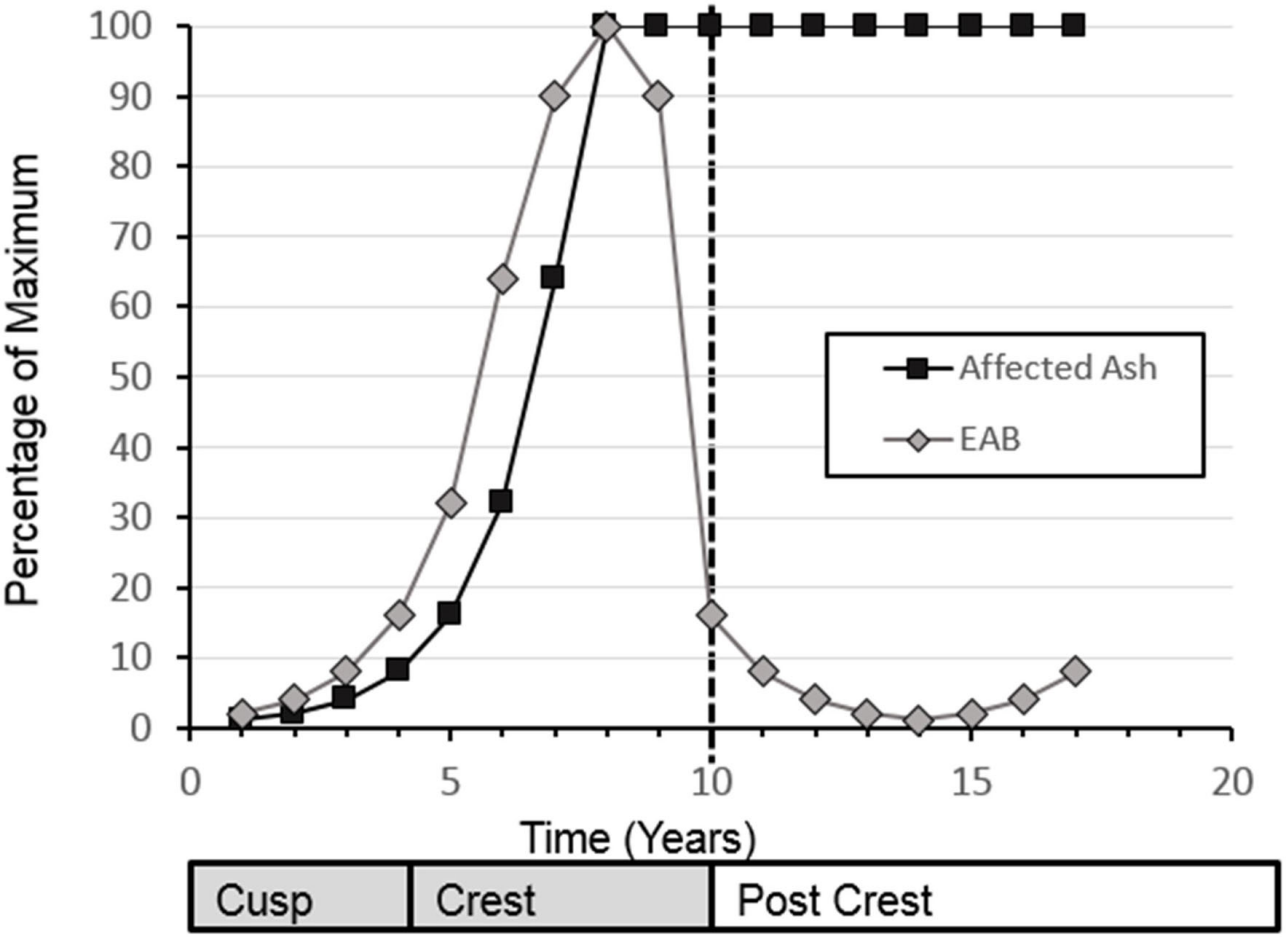
Conceptual diagram for emerald ash borer populations and impact on ash populations. Modified from Sadof.

EAB population dynamics after arriving to a new location follow a simple exponential curve for the growth phase of the invasion, followed by a sharp drop in EAB population after the peak of the outbreak (**Figure 1**). The increasing portion of the exponential curve model has been validated for tree health in three urban areas by Sadof et al. (6), and many studies that use tree health as a proxy for EAB levels show similar curves (7, 8). Longer-term empirical data on the EAB population levels that move beyond the exhaustion of the main ash source have been collected largely in the context of monitoring the impact of biological control agents (9, 10). Understanding what is happening with the EAB population itself is important for long-term planning of ash management and recovery. While the population crash of EAB at a site is attributed to decline of adult ash trees, better monitoring efforts would assist in management of secondary outbreaks co-occurring with the recovery of a new cohort of ash trees.

Landscape-scale spread of EAB has been an important question in modelling efforts to track the regional spread of this invasive insect. Typically, the pattern seen throughout the beetles’ history in North America is that satellite populations emerge as the result of anthropogenic activity leading to an unintentional introduction (11). While models of EAB movement have predicted a range of spread rates, non-anthropogenic dispersal of EAB is typically thought to 1 to 2 km a year after initial invasion, and to increases as the population of EAB increases (12–14). These sites then gradually coalesce with the larger invasive population. Similar regional patterns of spread have been shown through detection surveys such as the USDA APHIS purple prism trap survey (15). Improved or alternative monitoring techniques, like biosurveillance can help to validate these observations of EAB dispersal and establishment.

Biosurveillance for EAB, is conducted using *Cerceris fumipennis* (16). This solitary ground-nesting wasp uses adult buprestid beetles to provision their larvae. The wasp is native to eastern North America and is broadly found from Maine to Florida and west to Texas (17). Each female wasp digs and provisions her own nest, but nests are usually found in aggregations of tens to hundreds of individual withing the same area (18). Male wasps emerge first in mid-June, and mate with female wasps as they emerge from their underground nest cells. Female wasps dig burrows and once they have dug the initial brood cell begin to hunt. Females live an average of 18 days (19) but can survive up to 6 weeks. They sequentially dig cells for their young, filling each with sufficient beetle mass to support a larva through its development. The female wasp will dig, provision, and lay a single egg in as many cells as they can. The number of their offspring is limited by the rate at which they can dig and provision the cells before they die. The larvae then complete their development feeding on the paralyzed beetles and spin a cocoon (20). In the northern part of their range, including Connecticut, they overwinter as pre-pupae in their cocoons. Diapause is not obligate, and in the southern portions of its range, *C. fumipennis* has 2 generations a year (20) and infrequent second emergences have been observed in Connecticut (21).

Like many solitary wasps, *Cerceris fumipennis* preys on a single insect taxonomic group, adult beetles in the family Buprestidae. Over 100 species of buprestid beetles have been recorded as *Cerceris fumipennis* prey (22–25). In rare instances (< 0.1% of records) *Cerceris fumipennis* captures Cerambycidae and Chrysomelidae prey items (26). Otherwise, prey seems to be limited to buprestids that have an arboreal habitat as adults, are adults during the wasps’ hunting season, and are between 4-20 mm in length (27, 28).

Foraging wasps forage to maximize the mass of beetles collected for amount of effort (29). Wasps typically capture the most readily available prey rather than searching for specific prey species. Consequently, changes in the relative abundance of available prey are likely to be reflected in the abundance of species in the captured prey. Swink et al. (22) demonstrated changes in prey types for *C. fumipennis* in North Carolina during an outbreak of the hemlock borer, *Phaenops fulvuguttata* (Coleoptera: Buprestidae). Hemlocks stressed by *Adelges tsugae* (Hemiptera: Adelgidae) caused a proportional increase in hemlock borers as wasp prey. Changes in proportions of prey capture are demonstrated in other solitary hunting wasps as well including, *Hypodynerus andeus* (Packard) (30) and *Sphex ichneumoneus* (L.) (31).

In this paper we take advantage of data from 10 years of monitoring prey catch of *Cerceris fumipennis* (Hymenoptera: Crabronidae) to examine of the proportional abundance of EAB to other native buprestid insects among 112 sites in the state of Connecticut. These survey efforts have amassed a collection of over 30,000 individual buprestids that have been collected from forest fragments invaded by EAB. Due to high coverage within the state of Connecticut, these data can be used to map the spread of EAB in Connecticut since its introduction in New Haven Co., Connecticut. The aim of this study is to evaluate how informative the relative proportions of EAB in the prey of *C. fumipennis* colonies are for tracking EAB population levels. The resultant data examines the temporal history of the EAB infestation at single locations, as well as the spatial pattern of EAB distribution over time in Connecticut.

## 2 Materials and methods

### 2.1 Collection sites

Aggregations of *Cerceris fumipennis* were identified by surveys of baseball fields throughout Connecticut. Baseball fields are the most common habitat for the wasp where they aggregate in the sandy soils within foraging range of forests (18). In these surveys, new aggregations of *C. fumipennis* were added each year, and others were dropped as the numbers of wasps in colonies fluctuated.

The beetles were collected in June, July, and August from 2009-2022. At least 50 beetles per site per year was the goal for the survey, as Careless et al. (16) found that this number was a good compromise between detection of as many species of beetle as possible at a site, and available time. Beetles were collected by netting and releasing wasps carrying prey or by collecting beetles abandoned on the field by the wasps. (32, 33).

### 2.2 Proportion of EAB as related to time of EAB detection

For this analysis, a subset of sites that had greater than 30 beetles collected in the year before initial EAB detection and 50 beetles collected at the site for one or more years subsequent to the initial detection of Emerald Ash Borer at that site, were selected. For each site and year, the proportion of EAB/total beetles was calculated and classified by year relative to EAB detection, 0 = the year of detection, 1 = the year after detection, etc. regardless of what calendar year that detection occurred. 2012 provides the first records of EAB in these surveys, therefore data from 2009-2011 was not included in analyses. For hypothesis testing, we used a conceptual diagram as point of qualitative comparison between an expected curve of EAB populations (**Figure 1**) to our data collected.

#### 2.2.1 Modelling proportion of EAB against time of EAB detection

Proportion of EAB as a function of time of EAB detection was modelled using a generalized linear mixed effects model with a logit link. The full fixed effects model included time and time^2^, and the full random effects model included random slopes over time and time^2^ and random intercepts for collection site. Random effect selection was performed by comparing the full model to three simpler models (random intercepts and random slopes over time, random slopes over time only, and random intercepts only) based on (lowest) AIC (34). Modelling and model selection were conducted in R version 4.1.2 (35) using the lme4 and lmerTest packages, with model diagnosis as per Quinn & Keough (34). 95% confidence intervals were obtained using the ggeffects package version 1.1.4, and results were plotted using ggplot2 version 3.4.1.

### 2.3 Spatial analysis of EAB spread

For each year, starting in 2012 when EAB was first detected in Connecticut, we calculated the proportion of EAB in *C. fumipennis* prey by colony. Data from sites where less than 20 beetles were collected that year were excluded. We calculated Moran’s I to reveal significant spatial autocorrelation between all sites in each year (36). For visualizing the distribution of EAB proportional abundance in *C. fumipennis* colonies, we used Empirical Bayesian Kriging tool in ArcPro (36). In this approach, interpolated proportions between collection sites construct a series of maps, one per year, that show the density of EAB by color gradient.

## 3 Results

The first detection of emerald ash borer in Connecticut was made using *C. fumipennis* in 2012 in the town of Prospect, CT (33). Since 2012, EAB has been detected in all eight counties of Connecticut, and in 166 of its 169 towns. For three of those counties the first detection was made by *C. fumipennis*, as were 99 of the town detections (**Supplemental Data Figure 1**).

### 3.1 Proportion of EAB as related to time of initial EAB detection

**Figure 2:**
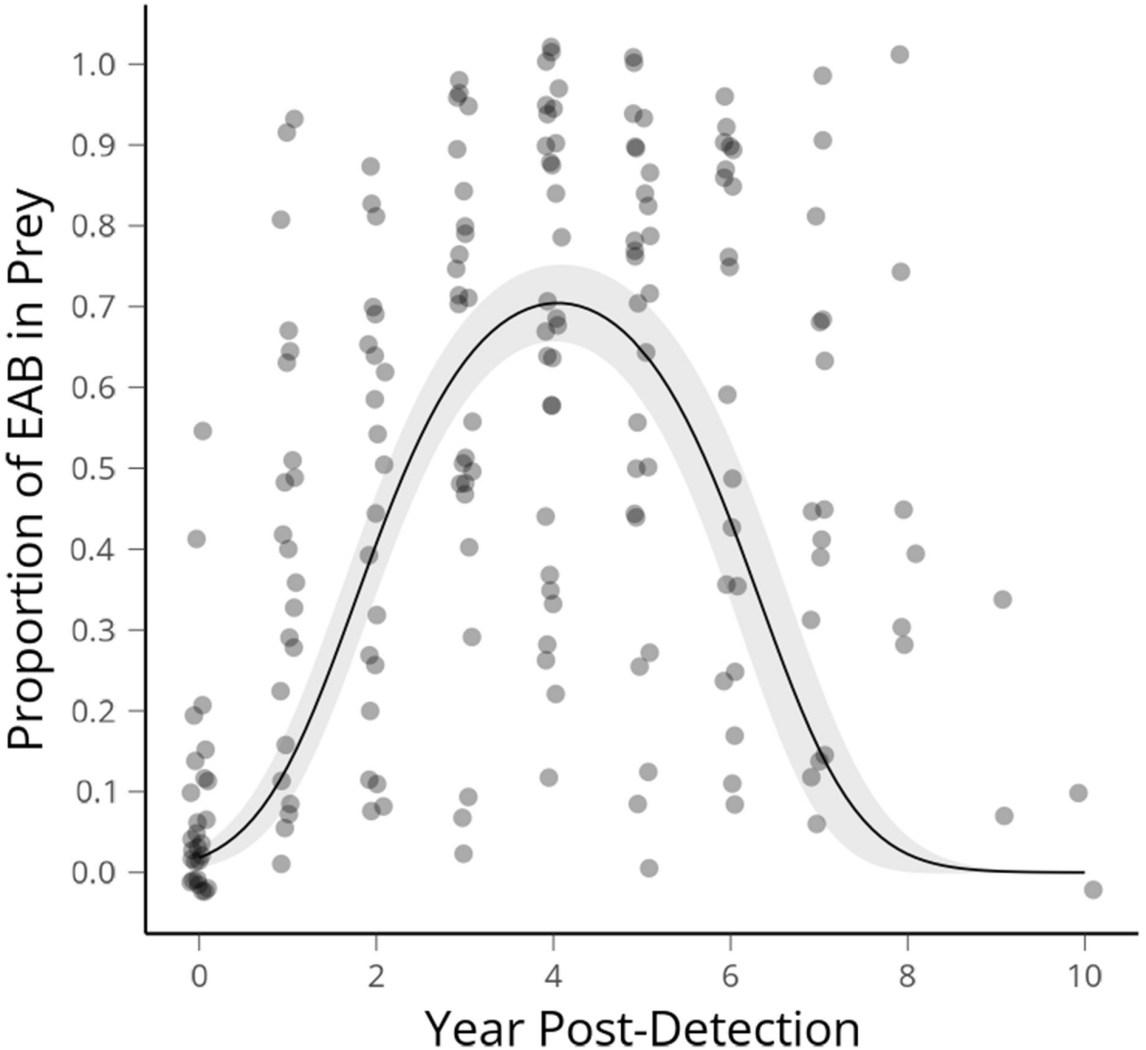
Average proportion of emerald ash borer in *Cerceris fumipennis* prey by year of detection, with the peak occurring at 4.05 years. The first year EAB is detected at a site is year 0, the next year 1 etc. Ribbon shows 95% confidence interval.

We had 48 sites for which we had a known first EAB detection year. This was defined as a site at which more than 30 beetles (X = 70.9 ± 5.25, Median = 66) were collected directly prior to the year EAB was first collected. Three sites which date from our first detections of EAB in 2012 reached the threshold of 10 years post-detection as of 2021, therefore encapsulating the range of time predicted for population rise and fall in wave front models (modified as conceptual diagram in **Figure 1**). Altogether 15,601 beetles are in the data set that was used to create this curve (**Figure 2**).

Average proportion of EAB in *C. fumipennis* prey predicted according to our GLMM had a peak occurring at 4.05 years. The span of arrival to population crash of EAB, as indicated by the proportional abundance of catches, spanned eight-ten years. The proportion of EAB collected in year zero was 0.108 (SE ± 0.026). If we assume the population starts at 1% and doubles each year, as in the theoretical model, that puts detection by *C. fumipennis*, on average, 4 to 5 years into the invasion. There was a range of EAB proportions from 0.01 – 0.52 in the first detection year. The mean proportion of EAB in *C. fumipennis* prey increased for 3 years after the initial detection. The proportion of EAB plateaued for the next 3 years, and then, starting 7 years after the initial detection, the proportion of EAB in wasp prey declined reaching an average of 0.108 (SE ± 0.07) by year 9.

### 3.2 Spatial analysis of EAB spread

**Figure 3:**
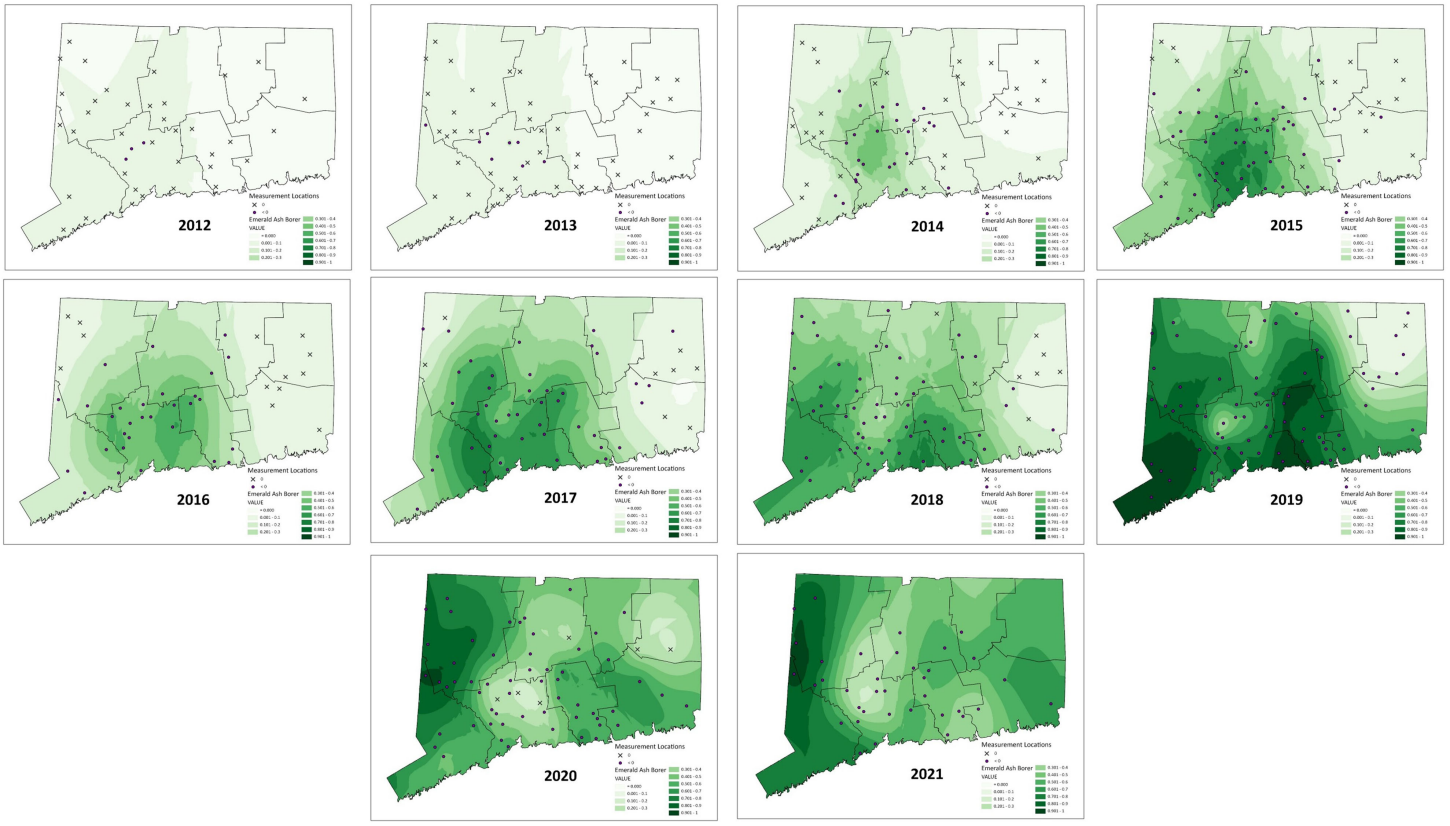
Maps of emerald ash borer density using the proportion of EAB in *Cerceris fumipennis* prey as a proxy. For each year, sites with over 20 beetles sampled that year are marked. Those marked with an ✕ are sites where no EAB were collected that year. Those marked with a purple circle represent sites where EAB was collected. The darker the green the higher the proportion of EAB found. Interpolation was done using Empirical Bayesian Kriging (36).

The number of sites on which the mapping was based in each year averaged 56 (SE ± 4.01, range 35 – 77). In 2020 – 2021 there were fewer sites surveyed due to difficulty recruiting volunteers during COVID quarantines, and unfavorable weather conditions. This was especially true in the north-eastern corner of the state. The resultant density predictions should be taken with caution for that part of the state in those years. Moran’s I, a measure of autocorrelation in spatial data, was significant in each year (**Table 1**), indicating that the data were good candidates for Empirical Bayesian Kriging. The models of EAB proportion across the landscape of Connecticut generated by Empirical Bayesian Kriging are shown in **Figure 3**. Statistics for Empirical Bayesian Kriging are available in the **Supplemental Data Table 1**.

**Table 1:** Moran’s Index of spatial autocorrelation for the proportion of emerald ash borer in *Cerceris fumipennis* prey by year.

Year	Moran’s I	*p*
2012	0.155	0.003
2013	0.100	0.001
2014	0.451	< 0.001
2015	0.639	< 0.001
2016	0.421	< 0.001
2017	0.528	< 0.001
2018	0.299	< 0.001
2019	0.519	< 0.001
2020	0.240	< 0.001
2021	0.361	< 0.001

## 4 Discussion

Detection of cryptic invasive species is critical for management. However, monitoring, especially monitoring that extends beyond initial detection, is seldom sustained long-term due to the difficulty of sampling. In this study we used *Cerceris fumipennis* prey capture as a proxy measurement for EAB presence and prevalence. This has allowed us to document both the temporal and spatial changes of EAB populations over the 10 years since EAB was detected in Connecticut.

Temporally, the rise of EAB as a proportion of prey captured by *C. fumipennis*, roughly mirrors the theoretical model. On average, the proportion of EAB in *C. fumipennis* prey was 8.7% in the first year of detection. This is consistent with detection at year 5 of the infestation and is the threshold at which infestations are commonly detected by trapping (6, 33). The proportion of EAB in *C. fumipennis* prey increased year over year, plateaued for 3 years, and then decreased. One striking difference when comparing the conceptual diagram of EAB populations to our results is the prolonged crest and gradual drop in EAB density. The theoretical curve drops steeply one year after the peak as it posits that all available ash has been exhausted. However, the sudden population drop seen in many eruptive forest pests, such as *Lymantria dispar dispar* (L.), is driven not only by decreases in food quality and availability, but by increasing predation by generalist predators, and by epizootics (37, 38). While food availability, and generalist predation, primarily by woodpeckers and generalist parasitoids of woodborers, are likely drivers of EAB population decline (39, 40), there is as-of-yet no evidence of epizootics impacting EAB in North America (41). The lack of specialized pathogens may explain the more gradual population descent seen in the *C. fumipennis*-based data.

Alternatively, the prolonged crest and gradual population decline seen in our data could be due to an averaging effect across many sites. There was variation between sites and years in these patterns and several factors that could be contributing to that variation. Ash is not distributed equally across the landscape. Although we did not test this explicitly it seems likely the density of ash in the 1.5 km foraging range of each *C. fumipennis* site (42) will impact both the peak proportion of EAB at the site, and the length of that peak. Another source of variation between sites is the timing of data collection each year. In Connecticut, *C. fumipennis* hunting season typically starts in mid-late June, with a peak during the first 2 weeks of July and lasting into August. Emerald ash borer, by contrast, typically emerges in early June and adult numbers tend to drop off by mid-July. Thus, sites for which a year’s data was collected in late July is likely to have a lower proportion of EAB in their catch than sites for which a year’s data was collected in early July. Finally, there were sites at which EAB was detected early or late in the infestation. For example, we had a site for which EAB had been detected in neighboring sites ringing it, but no EAB prey was detected at this site for 2 years. In contrast, we had sites for which EAB was detected one year, and it was then another 3 years before another EAB was caught. However, despite these factors, the aggregated data clearly reflect population patterns of EAB.

We were also able to obtain data on not only the spatial distribution of detection of EAB as it moved through the state, but its density at each site in each year it was sampled. We could then use empirical Bayesian kriging to generate maps to provide a clear picture of the invasion ‘wave’ as it moved across the state. The data clearly show the beetle spreading out in a radial fashion from its presumptive invasion site in northern New Haven County. The density dynamics are as expected, in each new area the density is at first low, and then quickly increases before gradually decreasing. Another pattern that is shown is that there was likely only one successful establishment of EAB in the state. At no point do we see loci of high EAB density that are not consistent with spread from the original detection area of northern New Haven County. This spatially explicit information can be used to assist managers to tailor EAB responses.

Future work will look toward understanding the movement we have seen in the light of more explicit landscape analysis. Factors to be investigated include impact of local ash density on rate of spread, and the presence of barriers to or corridors for EAB movement. Continued monitoring of *C. fumipennis* colonies is planned. Of particular interest is the dynamics of EAB populations in post-crest areas as the ash resource begins to recover.

## Data availability statement

The raw data supporting the conclusions of this article will be made available by the authors, without undue reservation.

## Author contributions

CR conceived of and designed of study. RC performed statistical analysis. CR wrote the first draft of the manuscript. RC wrote sections of the manuscript. All authors contributed to manuscript revision, read, and approved the submitted version.
