# Supplementary material for: Temporal and spatial dynamics of the emerald ash borer invasion in Connecticut as shown by the native digging wasp Cerceris fumipennis (Hymenoptera: Crabronidae)
Source: Front Insect Sci. 2023 May 15;3:1179368. doi: 10.3389/finsc.2023.1179368 (PMC10926490; doi:10.3389/finsc.2023.1179368)
Supplement: Supplementary file 1 [file DataSheet_1.docx]

Supplemental Data

Table 1. Statistics by year for Empirical Bayesian Kriging. Analysis was run in ArcPro (Esri 2022).

|  | 12 | 13 | 14 | 15 | 16 | 17 | 18 | 19 | 20 | 21 |
| --- | --- | --- | --- | --- | --- | --- | --- | --- | --- | --- |
| Count | 38 | 53 | 60 | 67 | 43 | 53 | 70 | 77 | 63 | 36 |
| Average CRPS | 0.011842 | 0.029207 | 0.062784 | 0.092723 | 0.124331 | 0.140422 | 0.157441 | 0.11934 | 0.14383 | 0.133792 |
| Inside 90 Percent Interval | 94.73684 | 98.11321 | 91.66667 | 89.55224 | 88.37209 | 88.67925 | 95.71429 | 93.50649 | 90.47619 | 94.44444 |
| Inside 95 Percent Interval | 94.73684 | 98.11321 | 93.33333 | 92.53731 | 95.34884 | 96.22642 | 98.57143 | 94.80519 | 95.2381 | 97.22222 |
| Mean | 0.003079 | 0.006615 | -0.00349 | 0.004463 | 0.014745 | 0.015735 | 0.008352 | -0.00018 | -0.00393 | -0.01598 |
| Root-Mean-Square | 0.025506 | 0.071365 | 0.120017 | 0.167545 | 0.219184 | 0.24788 | 0.275509 | 0.212609 | 0.25388 | 0.229482 |
| Mean Standardized | 0.11477 | 0.090324 | -0.02315 | 0.01353 | 0.049474 | 0.047991 | 0.023972 | 0.004329 | -0.01325 | -0.04676 |
| Root-Mean-Square Standardized | 0.980368 | 0.986514 | 0.897049 | 0.968091 | 0.963086 | 0.985122 | 0.999567 | 0.957663 | 0.999082 | 0.954591 |
| Average Standard Error | 0.026372 | 0.073224 | 0.149662 | 0.182537 | 0.23601 | 0.256197 | 0.273613 | 0.225082 | 0.252313 | 0.249121 |

Supplemental Data – Table 2. Logistic generalized linear mixed model for the proportion of EAB as a function of time. Z-scores and *p* values are specified for fixed effects, and log Likelihood and AIC are specified for random effects (in italics). The best random effects structure was selected based on (lowest) AIC. Significant terms are highlighted in bold.

| **Term** | **Z value/*logLik*** | **p/*AIC*** |
| --- | --- | --- |
| **Fixed Effects** |  |  |
| Time | 6.68 | **< 10^-10^** |
| Time^2^ | -6.31 | **< 10^-9^** |
| ***Random Effects*** |  |  |
| *(1 + Time + Time^2^ \| Site)* | *-1121* | ***2261*** |
| *(1 + Time \| Site)* | *-1443* | *2899* |
| *(Time \| Site)* | *-1443* | *2899* |
| *(1 \| Site)* | *-2083* | *4173* |

Supplemental Data - Figure 1. A map of emerald ash borer detection in the state of Connecticut by year. Stars indicate towns where the first detection of emerald ash borer was made by monitoring *Cerceris fumipennis* prey. The presence of emerald ash borer in 3 of the 169 towns remains officially unconfirmed, but it is assumed.

N

2012

County Boundary

Town Boundary

2013

2014

2015

2016

2017

2018

2019

2020

2021

1^st^ Detection by

*Cerceris fumipennis*

Emerald Ash Borer First Detected
